# Prevalence of burnout syndrome in clinical nurses at a hospital of excellence

**DOI:** 10.1186/1755-7682-7-22

**Published:** 2014-05-09

**Authors:** Vivian F Ribeiro, Celso Ferreira Filho, Vitor E Valenti, Marcelo Ferreira, Luiz Carlos de Abreu, Tatiana Dias de Carvalho, Valdelias Xavier, JapyAngeli de Oliveira Filho, Pedro Gregory, Eliseth Ribeiro Leão, Natascha G Francisco, Celso Ferreira

**Affiliations:** 1 Departamento de Medicina, Disciplina de Cardiologia, UNIFESP, Rua Sena Madureira, 1500 - 5° andar, São Paulo, SP 04021-001, Brazil; 2 Departamento de Fonoaudiologia, Faculdade de Filosofia e Ciências, UNESP, Av. HyginoMuzziFilho, 737, Marília P 17525-900, Brazil; 3 Departamento de Morfologia e Fisiologia, Faculdade de Medicina do ABC, Av. Príncipe de Gales, 821, Santo André, SP 09060-650, Brazil

**Keywords:** Burnout, Professional, Syndrome, Nursing

## Abstract

**Background:**

Burnout syndrome can be defined as long-term work stress resulting from the interaction between constant emotional pressure associated with intense interpersonal involvement for long periods of time and personal characteristics. We investigated the prevalence/propensity of Burnout syndrome in clinical nurses, and the factors related to Burnout syndrome-associated such as socio-demographic characteristics, work load, social and family life, leisure activities, extra work activities, physical activities, and work-related health problems.

**Method:**

We conducted a cross-sectional, quantitative, prospective epidemiological study with 188 surgical clinic nurses. We used the Maslach Burnout Inventory (MBI), which is a socio-demographic questionnaire and the most widely used instrument to assess Burnout syndrome (three basic dimensions: emotional exhaustion, despersonalization and professional underachievement). The socio-demographic profile questionnaire wascomposed of questions regarding identification, training, time at work, work characteristics and personal circumstances.

**Results:**

The prevalence of Burnout syndrome was higher (10.1%) and 55, 4% of subjects had a propensity to develop this syndrome. The analysis of the socio-demographic profile of the nurse sample studied showed that most nurses were childless married women, over 35 years of age, working the day shift for 36 hours weekly on average, with 2-6 years of post-graduation experience, and without extra employments. Factors such as marital status, work load, emotion and work related stress aggravated the onset of the syndrome.

**Conclusion:**

The prevalence and propensity of Burnout syndrome were high. Some factors identified can be useful for the adoption of preventive actions in order to decrease the prevalence of the clinical nurses Burnout syndrome.

## Background

Nurses are commonly exposed to stress due to work overcharge [1-3]. In this context, Burnout syndrome can be defined as long-term work stress resulting from the interaction between constant emotional pressure associated with intense interpersonal involvement for long periods of time and personal characteristics. Frequent Burnout syndrome symptoms include emotional exhaustion and development of negative attitudes and feelings towards work colleagues as well as to their own professional achievement [4].

Even though, Burnout syndrome is detected in professionals from various areas, prevalence is particularly high in service and care workers, especially health and care ones [5]. Among those, nurses have been the subject of several studies, because they experience constant stressful labor situations, working in direct contact with patients who have different expectations and degrees of suffering. For instance, a study conducted in Europe in 2011 showed that approximately 30% of nurses surveyed reported being exhausted or fatigued due to work activities [6,7]. In addition, a British study found that approximately 42% of nurses in England reported to be suffering from Burnout, whereas in Greece approximately 44% of nurses reported a feeling of dissatisfaction at work and a desire to leave work. Lower prevalence was reported in a survey in Germany, which estimated that 4.2% of that worker population was affected by Burnout [6-8].

However, few studies in Brazil have investigated only nurses. In most cases, the number of professionals in the institutions investigated is relatively small, leading to the joint study of nurses, technicians, and assistants, whose professional activities differ in nature, complexity, and emotional overload. Thus, it is difficult to determine the exact prevalence of this condition among nurses in Brazil. Moreover, studies on Burnout syndrome in Brazil have largely overlooked high-quality institutions that constantly strive to obtain health care quality certifications. Thus, it is not known, for instance, whether the prevalence of the syndrome among nurses in high-quality institutions differs from that observed in other institutions. That knowledge is necessary to identify factors associated with the onset of the syndrome and to develop plans for prevention and control.

In this study, we investigated the prevalence/propensity of Burnout syndrome in clinical nurses, and the factors related to Burnout syndrome associated, such as socio-demographic characteristics, work load, social and family life, leisure activities, extra work activities, physical activities, and work-related health problems. Nurses with and without Burnout syndrome, or Burnout propensity were compared.

## Methods

We conducted a cross-sectional, quantitative, prospective epidemiological study with 188 surgical clinic nurses. The study was conducted from August to October 2012. One individual refused to participate in the study, and twelve nurses were on sick leave. The study site is a large private hospital in the city of São Paulo, considered the best hospital in Latin America and a benchmark in quality and excellence [9].

We used the Maslach Burnout Inventory (MBI), which is a socio-demographic questionnaire and the most widely used instrument to assess Burnout syndrome [10].

The MBI assesses how workers experienced their work, according to three conceptual dimensions: emotional exhaustion, depersonalization and personal accomplishments. The most widely used version of the MBI was established in 1986, it began to be used only for the evaluation of the frequency, since the existence of high correlation between the two scales was detected, and many studies found higher correlation (r = 0.80) [11-13].

The internal consistency of the three dimensions of the inventory is satisfactory, as it has a Cronbach’s alpha ranging from 0.71 to 0.90 and test-retest coefficients ranging from 0.60 to 0.80 in periods of up to one month [14].

In Brazil there were several attempts to translate, adapt and validate the MBI, since the researchers were concerned with overcoming the limitations of the low internal consistencies of the factors depersonalization and personal accomplishments, however, the MBI has been applied in more than 90% of the studies on Burnout worldwide [15]. According to some authors, there was success in the development of MBI as a tool for the assessment of Burnout in Brazil as indices of reliability Alpha Cronbach’s of 0.8014 [16-18].

The Maslach Burnout Inventory (MBI) assesses three basic dimensions: emotional exhaustion (low: scores lower than 19; intermediate: 19–26; and high: scores greater than 27); depersonalization (low: scores lower than 6; intermediate: 6–9; and high: scores greater than 10); and professional underachievement, which is inverse to the former two dimensions (low: scores greater than 40; intermediate: 34–49; and high: scores lower than or equal to 33) [19]. The socio-demographic profile questionnaire is composed of a set of close-ended, multiple-choice, and open-ended questions, covering different spheres of life of the subject: identification (sex, marital status, age, children), training, time at work, work characteristics (employment status, time at work, other jobs, number of working hours per day, shift, weekly rest, work impressions, pleasant and unpleasant work attributes), personal circumstances (work related health problems, non-work related activities, dedication to family on weekends, symptoms, and a self-assessment on stress).

The questionnaires were administered after nurses had signed the informed consent form and approved by the Ethical Committee in Research (CAAE n° 04793112.4.3001.0071).

The data were described by absolute frequencies and percentages. The comparisons between genders and work shifts were performed using Pearson’s chi-square test or Fisher’s exact test in cases where expected frequencies were lower than five. The analyses were performed using SPSS statistical software (SPSS Inc. Released 2008. SPSS Statistics for Windows, Version 17.0. Chicago: SPSS Inc.). Significance level was set at 5%.

## Results

### High prevalence of Burnout syndrome

**Figure 1:**
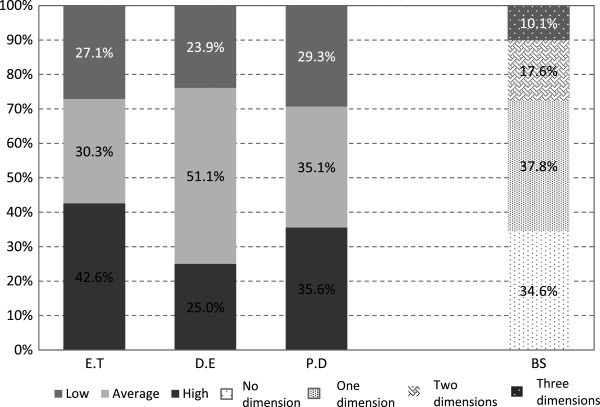
**Burnout syndrome dimensions in clinical nurses from August to October 2012 at Albert Einstein Jewish Hospital, São Paulo, Brazil.** ET = Emotional tiredness. DE = Depersonalization. PD = Professional underachievement. BS = Burnout syndrome.

The detection of the three dimensions that characterize Burnout syndrome (concurrent emotional tiredness, depersonalization, and professional underachievement) or of one or two of those dimensions (indicative of predisposition to the syndrome) is shown in Figure 1. Approximately 10% of the studied population had Burnout syndrome, and over half the nurses interviewed had a propensity to develop the syndrome (presence of one or two dimensions). We observed the prevalence, in decreasing order, of high emotional exhaustion, professional underachievement, and depersonalization.

The analysis of the socio-demographic profile of the nurse population studied showed that most nurses were childless married women, over 35 years of age, with knowledge of Burnout syndrome, working the day shift and 36 hours weekly on average, with 2-6 years since graduation, and without additional employments (Table 1).

**Table 1:** Socio-demographic characteristics of clinical nurses from August to October 2012 at Albert Einstein Jewish Hospital, São Paulo, Brazil

**Variables**	**n (%)**
**Sex**	
*Man*	31 (16)
*Woman*	137 (83)
**Age**	
*20 a 25*	17 (9)
*26 a 30*	54 (29)
*31 a 35*	55 (29)
*35+*	62 (33)
**Work shift**	
*Day*	111 (59)
*Night*	77 (41)
**Marital status**	
*Married*	103 (55)
*Single*	71 (38)
*Separated*	3 (2)
*Divorced*	10 (5)
*Widowed*	1 (0.5)
**Children**	
*Yes*	84 (45)
*No*	104 (55)
**Time since graduation (years)**	
*< 2*	17 (9)
*2–6*	80 (43)
*7–9*	73 (39)
*> 20*	18 (10)
**Work hours**	
*36*	172 (92)
*> 36*	16 (9)
**The nurse has other employment**	
*Yes*	15 (8)
*No*	173 (92)

Table 2 also shows that most subjects interviewed did not have other activities other than work, with no or rare weekends devoted exclusively to family, leisure activities, and social life. Regarding the prevalence of work-related diseases, most subjects reported having no problems. Among the subjects who reported having health problems, the main work-related health complaints were musculoskeletal pain and emotional complaints.

**Table 2:** Extra-work activities, health problems, and health complaints reported by clinical nurses from August to October 2012 at Albert Einstein Jewish Hospital, São Paulo, Brazil

**Variables**	**n (%)**
Extra-work activities	
*YES*	87 (46)
*NO*	101 (54)
Which ones?	
Sport	36 (41)
Leisure	6 (7)
Voluntary and religious activities	4 (2)
Home	18 (20)
Education	28 (30)
Other	11 (13)
Health problems	
YES	50 (27)
*NO*	136 (73)
Work related health complaints	
Musculoskeletal pain	24 (48)
Emotional complaints	20 (40)
Headache	7 (14)
Other	7 (14)
Did not answer	1 (2)

### Comparisons between subjects with Burnout propensity and Burnout syndrome and subjects without Burnout syndrome

According to Table 3 the simultaneous presence of the three dimensions (emotional exhaustion, depersonalization, and professional underachievement) indicates the occurrence of Burnout syndrome, whereas the presence of one or two dimensions indicates a propensity to develop the syndrome, which has a great impact on the work activity and worker life. The proportion of women was higher in subjects with Burnout propensity than in subjects without the syndrome, but no significant differences were observed between the two groups (p = 0.169).

**Table 3:** Comparisons between subjects with Burnout propensity and Burnout syndrome and subjects without Burnout syndrome and demographiccharacteristics, work aloud and health problems from August to October 2012 at Albert Einstein Jewish Hospital, São Paulo, Brazil

	**Dimension classification**							
	**No dimension**		**Propensity**		**P**	**Burnout**		**p**
	**N**	**%**	**N**	**%**		**N**	**%**	
**Sex**								
Man	14	45,2	14	45,2	0,169	3	9,7	0,751
Woman	51	32,5	90	57,3		16	10,2	
**Work shift**								
Day	46	41,4	55	49,5	**0,021**	10	9,0	0,140
Night	19	24,7	49	63,6		9	11,7	
**Age**								
20 a 25 yrs	4	23,5	9	52,9	0,290	4	23,5	0,249
26 a 30 yrs	15	27,8	35	64,8		4	7,4	
31 a 35 yrs	19	34,5	30	54,5		6	10,9	
➢ 35 yrs	27	43,5	30	48,4		5	8,1	
**Marital status**								
Married	41	39,8	53	51,5	**0,007**	9	8,7	0,327
Single	15	21,1	47	66,2		9	12,7	
Separeted	2	66,7	1	33,3		0	0,0	
Divorced	6	60,0	3	30,0		1	10,0	
Widove	1	100,0	0	0,0		0	0,0	
**Children**								
No	32	30,8	60	57,7	0,283	12	11,5	0,285
Yes	33	39,3	44	52,4		7	8,3	
**Weekly hours**								
36 hrs	56	33,3	93	55,4	0,995	19	11,3	0,327
Others workload	6	37,5	10	62,5		0	0,0	
**Weekends are devoted exclusively to family, social life and lesure activities**								
Never	1	25,0	2	50,0	0,411	1	25,0	0,089
Rarely or few times	30	29,1	59	57,3		14	13,6	
Several times or almost always	19	38,8	28	57,1		2	4,1	
Always	15	46,9	15	46,9		2	6,3	
**Others employment**								
No	58	33,5	98	56,6	0,235	17	9,8	>0,99
Yes	7	46,7	6	40,0		2	13,3	
**Extra- work activities**								
No	41	40,6	46	45,5	**0,017**	14	13,9	0,392
Yes	24	27,6	58	66,7		5	5,7	
**Performance of physical activities**								
No	12	23,5	35	68,6	0,389	4	7,8	0,343
Yes	12	33,3	23	63,9		1	2,8	
**Work- related health problems**								
No	55	40,4	72	52,9	**0,038**	9	6,6	**0,002**
Yes	10	20,0	30	60,0		10	20,0	
**Pain**								
No pain complaint	60	37,3	87	54,0	0,380	14	8,7	0,073
One pain complaint	3	17,6	11	64,7		3	17,6	
Two pain complaints	2	25,0	4	50,0		2	25,0	
Three pain complaints	0	0,0	0	0,0		0	0,0	
**Emotional complaints**								
No emotional complaints	64	38,6	88	53,0	**0,022**	14	8,4	**0,002**
One emotional complaint	1	7,7	10	76,9		2	15,4	
Two or more emotional complaints	0	0,0	4	57,1		3	42,9	
**Education activities**								
No	19	31,7	39	65,0	0,280	2	3,3	0,112
Yes	5	18,5	19	70,4		3	11,1	
**Home activities**								
No	21	30,4%	44	63,8	0,370	4	5,8%	0,553
Yes	3	16,7	14	77,8		1	5,6	

Propensity = 1 or 2 dimensions.

Burnout syndrome = 3 dimensions.

Propensity = 1 or 2 dimensions.

Burnout syndrome = 3 dimensions.

Bold is significant.

The proportion of subjects who work the night shift was higher in subjects with Burnout propensity than in subjects without the syndrome (p = 0.021).

The proportion of singles was higher in subjects with a propensity to develop the syndrome than in subjects without the syndrome (p = 0.007).

The proportion of nurses who have extra-work activities was higher in subjects with Burnout propensity that in subjects without the syndrome (p = 0.017).

The number of emotional complaints was higher in subjects with Burnout syndrome (p = 0.002) and Burnout propensity (p = 0.022) than in subjects without the syndrome.

The proportion of nurses with work related health problems was higher in subjects with Burnout than in those without the syndrome (p = 0.002).

As expected, the frequency of work related health problems differed significantly among the number of dimensions identified and was greater in subjects with Burnout syndrome. It should be noted that approximately 40% of subjects who reported having no health problems also showed no Burnout dimensions. Similarly, and as expected, we found a significant positive association between the number of emotional complaints and the incidence of Burnout (p= 0.038).

The number of subjects who perform physical activities was higher in subjects with Burnout propensity than in subjects without the syndrome, but no significant differences were observed (p = 0.389).

The number of subjects who never dedicate their weekends to family, leisure activities, and social life was higher in subjects with Burnout propensity than in subjects without the syndrome, but no significant differences were observed (p = 0.411).

## Discussion

This study aimed to evaluate the prevalence of Burnout syndrome in a large sample of clinical nurses and possible socio-demographic factors and activities associated with the syndrome. Our results indicate that the prevalence of Burnout syndrome is high in this sample, which was predominantly composed of childless, women without additional employments, who rarely or occasionally dedicated weekends to family, leisure activities, and social life. Moreover, most subjects did not participate in educational or sport activities in addition to work, and reported having frequent health and emotional complaints.

This is the first study to establish the prevalence of Burnout syndrome in a group of Brazilian population composed exclusively of clinical nurses working in a hospital of renowned quality, with quality certifications for nursing professionals. Thus, our results should be useful for defining the behavior of the syndrome and possible changes in prevalence due to the adoption of specific prevention measures. Those measures should be beneficial not only to the individuals affected, but also to their health institutions as Burnout syndrome directly affects healthcare organizations, harming the quality of services and increasing the rate of dissatisfaction and absenteeism.

The prevalence of Burnout in this study was10.1% higher than in previous studies conducted in Brazil that range from 0 to 4.7% [8-10] and 49.7% in international studies [16-18].

One possible explanation for this difference is the heterogeneity of the sample population in previous studies, which included not only nurses, but also technicians and assistants. Because of the differences in work functions, in addition to the fact that they work at different hospital areas, the prevalence of Burnout may also differ among these professionals, negating a direct comparison with prevalence rates observed in this study, whose sample consisted of clinical nurses only.

It should also be noted that the nurses at the institution evaluated perform several activities after work hours and have to attend 53 hours of courses annually, in addition to participate in congresses and intra-sectoral audits.

This accumulation of tasks may determine the short time devoted to family, leisure activities, and social life, resulting in dissatisfaction, feelings of guilt and helplessness, reducing self-esteem and, at a more advanced stage, professional achievement.

The prevalence rate of Burnout was 10,1% in our sample population, our results also showed that more than half of the interviewees (55,4%) had a propensity to develop Burnout syndrome, especially among single, childless subjects.

The fact that most of the sample population was composed of women over 35 years of age may have influenced this result, because of the possible occurrence of feelings of inferiority and frustration in light of current cultural standards that still consider married women with children to be happier and more complete. Moreover, the lack of support and comfort from family may contribute to excessive dedication to work, thus increasing the propensity to Burnout.

Our results also indicate that the frequency of emotional and health (mainly musculoskeletal pain) complaints was, as expected, higher in individuals affected by the syndrome. These findings are consistent with the high rates of sick leave associated with Burnout, and confirm the expectation that the emotional stress generated by the complexity of nursing work and the direct contact with patients who experience different degrees of suffering and have different expectations can lead to emotional overload in these professionals [19,20].

We also observed that the propensity to develop the syndrome was higher among nurses working the night shift – an expected result considering that these professionals have no regular sleeping and eating patterns. Moreover, feelings of inadequacy may be present, because while everyone else is working, this professional sleeps, performing his/her activities outside the regular routine adopted by society. Thus, the creation of flexible work schedules and measures to adopt a different policy for professionals who work at night could help reduce the prevalence of the syndrome.

Regarding the factors that may help prevent Burnout such as physical activity, we observed no significant differences in the propensity (or prevalence) of Burnout between nurses who performed and did not perform such activities. Even though physical activity is associated with numerous health benefits, it can become another burden for individuals burdened with other work activities.

It is not possible to establish a causal relationship between the frequency of sports practice and the prevalence of Burnout because of the retrospective nature of our study and the possibility that this factor may interact with other factors that were not analyzed. Thus, we believe that sports should be encouraged in a healthy way, so that they can be recognized as a source of pleasure and satisfaction.

We also observed that a higher frequency of educational and home activities were associated with the propensity to develop the syndrome. This result is consistent with the notion that such activities could represent an additional study and work overload for these professionals, negatively affecting – at least in the short term – their physical and emotional health. Thus, individuals who also study and manage their homes may not perceive the positive aspects of these additional activities, and consider their work stressful.

This study has some limitations. Even though the onset of Burnout is a consequence of the interaction between stress and constant work overload and personal, lifestyle, and personality traits, this study did not evaluate individual variables such as how subjects perceive work and their expectations towards work, as well as personal and personality traits that tend to prevent Burnout. Thus, future studies that evaluate such characteristics may provide subsidies for the development of prevention strategies that consider both institutional measures and personal and lifestyle interventions to be made by each professional.

## Conclusion

The prevalence and propensity for Burnout syndrome was higher specially among women, single and childless subjects. Regarding the individuals who suffer from Burnout syndrome a direct connection was made to health problems related to work. Individuals who did not have any dimension did not report any health problems associated with work.

Working during night, being single and developing activities outside the work was present in individuals who were prone to the syndrome.

Emotional complaints were present in both: Burnout and propensity.

Physicaly active female individual who dedicated time to family and social life were equally as prone to the condition as those who did not and showed no significant statistical difference.

## Competing interests

The authors declare that they have no competing interests.

## Authors’ contributions

All authors participated in the acquisition of data and revision of the manuscript. All authors determined the design, interpreted the data and drafted the manuscript. All authors read and gave final approval for the version submitted for publication.
